# Validating ground-based aerodynamic levitation surface tension measurements through a study on Al_2_O_3_

**DOI:** 10.1038/s41526-022-00213-8

**Published:** 2022-07-19

**Authors:** Yifan Sun, Guangtao Duan, Akifumi Yamaji, Tomoya Takatani, Hiroaki Muta, Yuji Ohishi

**Affiliations:** 1 Graduate School of Engineering, Osaka University, Osaka, Japan; 2 Department of Nuclear Engineering and Management, Graduate School of Engineering, The University of Tokyo, Tokyo, Japan; 3 Graduate School of Advanced Science and Engineering, Waseda University, Tokyo, Japan

**Keywords:** Characterization and analytical techniques, Fluid dynamics

## Abstract

The surface tension of a molten sample can be evaluated based on its resonant frequency with various levitation techniques. Under a 1-G condition, the use of levitation forces to counteract gravity will cause the levitated sample’s resonant frequency to differ from that under microgravity. A mathematical relationship to correct for this deviation is not available for a sample levitated with aerodynamic levitation (ADL), which raises issues on the validity of surface tension measurements done with ADL. In this study, we compared the surface tension of molten Al_2_O_3_ obtained using the front tracking (FT) simulation method, the drop-bounce method with ADL, and the oscillating drop method with ADL. The drop-bounce method simulates microgravity by allowing the sample to free-fall over a period of tens of milliseconds. Based on the results of this comparison, we determined that the surface tension of molten materials measured with ground-based ADL with the oscillating drop method, calculated using the resonant frequency of the l=2 m=0 mode, only shows a small deviation from that obtained under microgravity.

## Introduction

The thermophysical properties of molten materials, such as its surface tension, give us control of the structure and properties of the solid upon cooling. Conventional contact techniques such as the sessile/pendant drop method^1–6^ and maximum bubble pressure method^7–9^, for surface tension measurements have been used to investigate these properties of high-temperature molten materials. However, various issues at elevated temperatures, such as sample contamination, temperature inhomogeneity, calibration difficulties have resulted in a dearth of reliable experimental data^10^. With the development of levitation techniques such as electrostatic levitation (ESL), electromagnetic levitation (EML), and aerodynamic levitation (ADL), the concern of high-temperature molten samples reacting with the container has been eliminated. In addition, the contact-less environment can inhibit the sample’s heterogeneous nucleation and allow the properties of deep-undercooled liquids to be studied^11–13^.

Oscillation techniques, where a levitated sample’s resonant frequency is measured, have been used with ESL, EML, and ADL to determine the surface tension of molten materials^14–16^. For EML, the sample’s oscillation is spontaneous, whereas for ESL and ADL, oscillation needs to be manually introduced. It is preferable to conduct these oscillating drop experiments under microgravity because a wider range of materials can be levitated, issues such as evaporation can be avoided, and data with greater accuracy can be collected compared to ground-based experiments^17–19^. These advantages of performing the oscillating drop method in microgravity arise from the fact that sample levitation can be achieved without the need for strong levitation forces to counteract the effect of gravity. These external forces will deform the levitated sample’s shape and rotate the sample, resulting in a shift and split in the sample’s resonant frequency, which are commonly observed in ground-based EML experiments^17^. For a rotating aspherical sample, the degeneracy of the l = 2 mode’s resonant frequency is lost and a total of five characteristic frequencies (l = 2 m = 0, ±1, ±2) are observed^17^. For ESL, the sample is almost spherical, and its rotation is carefully controlled to suppress unwanted oscillation modes and minimize deviations from the characteristic oscillation frequency^20^. For ADL, such splitting can be observed but one can use smaller samples to reduce sample deformation during levitation^16^.

The Rayleigh equation^21^ is used to calculate the sample’s surface tension with the oscillating drop method. Because the Rayleigh equation assumes that the sample is force-free, non-rotating, and spherical, it should only be used in surface tension calculations if the oscillating drop experiments are performed under microgravity. However, ground-based experiments are much more accessible and less costly, which has motivated many researchers^22–24^ to modify the Rayleigh equation so that it can be applied to a sample levitated under 1-G. The validity of these modifications is then confirmed with microgravity experiments^10,20,22,23,25^. For ground-based EML, because the levitated sample is heavily deformed, the Cummings–Blackburn’s sum-rule^23^ is always applied prior to using the Rayleigh equation for surface tension calculations. In contrast with the availability of corrections terms for ESL and EML under 1-G, there are no similar theoretical corrections available for ADL—which specializes in the levitation of oxides. Such a correction requires one to clarify the force acting on the aerodynamically levitated droplet, which is not readily available^16^. Therefore, it is currently unknown how the oscillation characteristics of a droplet levitated with ground-based ADL differ from that of a freely oscillating droplet. If such a relationship can be established, ground-based ADL can provide surface tension data of molten oxides that will contribute greatly to fields such as inorganic glass, thermal/electrical insulators, and nuclear engineering.

To investigate the connection between the oscillation characteristics of a droplet under microgravity and during oscillating drop experiments with ADL, in this study, we combined the front tracking (FT) method and the drop-bounce method from our previous work^26^. The FT method is a well-established method for accurately simulating the large deformation of droplets or bubbles^27^. The drop-bounce method uses the sample’s oscillation pattern during free-fall to calculate its surface tension and, in our previous work^26^, we showed that the obtained data of liquid gold matched well with reference data obtained under microgravity^25^. With the FT method and the drop-bounce method, we addressed the question of whether corrections to the resonant frequency, like the Cummings–Blackburn’s sum-rule when calculating surface tension, is necessary for ADL in the following steps:

- Perform FT simulation to show the oscillation of a freely levitated sample (microgravity) and a bouncing sample (1-G) are identical.
- Use the drop-bounce method to evaluate the surface tension of molten Al_2_O_3_. Al_2_O_3_ was chosen here because Langstaff et al.^16^ performed oscillating drop experiments on molten Al_2_O_3_ with ADL under the 1-G condition and calculated the surface tension using only the resonant frequency of the l = 2 m = 0 mode. They also interpreted their ADL data using EML’s sum-rule.
- Compare the obtained surface tension value using the drop-bounce method with that calculated by Langstaff et al.^16^ using just the resonant frequency of the l = 2 m = 0 mode.

Based on the FT simulation results and by comparing the surface tension of molten Al_2_O_3_ obtained with the drop-bounce method to that reported by Langstaff et al.^16^, we determined whether a “sum-rule" is necessary to correct for the spilt in the sample’s resonant frequency during the oscillating drop experiment with ADL under the 1-G condition.

## Results

### Droplet oscillation behaviors simulated with the front tracking method

**Fig. 1:**
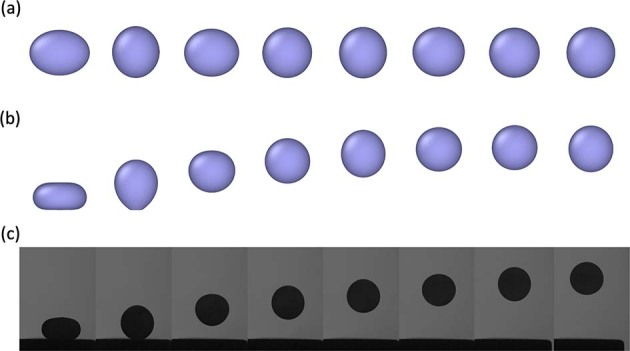
Oscillation behaviors of molten Al_2_O_3_ samples. **a** shows the result of a simulated freely-levitated oscillating molten Al_2_O_3_ sample. **b** is the simulated result of a molten Al_2_O_3_ sample that is bouncing upward after being dropped. **c** shows the behavior of a molten Al_2_O_3_ sample bouncing upward during the drop-bounce experiment. The photographs in **c** were taken by T. Takatani.

**Fig. 2:**
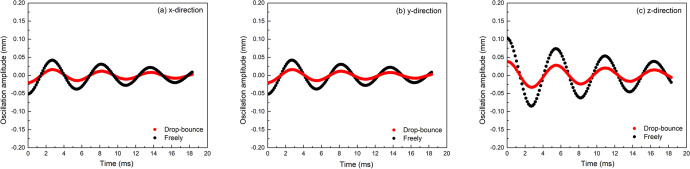
Comparisons between the droplet’s oscillation patterns in the x, y, and z directions in the two simulated scenarios. **a** shows the oscillations along the *x*-axis, **b** shows the oscillations along the *y*-axis, and **c** shows the oscillations along the vertical *z*-axis. For the simulated freely-levitated oscillating Al_2_O_3_ sample, its oscillation amplitudes are shown as black circles. The red circles represent the oscillation amplitudes of the simulated Al_2_O_3_ sample while bouncing upward. The Al_2_O_3_ samples have essentially the same oscillation pattern under these two simulated scenarios, with the only difference being the oscillation amplitude.

To simulate the droplet oscillation by computational fluid dynamics (CFD), we needed to specify the required physical properties, such as density, surface tension, and viscosity, which are the input parameters. The output surface tension is then evaluated using Eq. (2), based on the simulated oscillation behaviors of a droplet. The thermophysical parameters of the simulated droplet (pseudo molten Al_2_O_3_) used in the FT analysis are listed in Table 1. In the simulation, the simulated sample was assumed to be at its melting point and the influence of temperature on surface tension was not considered. Specifically, the surface tension was regarded as a constant at the melting point, and the heat transfer was not considered. It should be noted that gravity is not considered in the freely oscillating case but considered in the drop-bounce case. The simulated oscillation of a freely levitated droplet and that of a bouncing droplet are shown as a series of images in Fig. 1. The changes in the ellipsoid-shaped sample’s radius along the x, y, and z directions with time can be used to generate the oscillation patterns shown in Fig. 2. The x and y directions are along the horizontal plane whereas the z direction is along the vertical axis. As a l = 2 mode oscillation was induced in the simulation, its resonant frequency can be calculated by fitting the sample’s oscillation curves in Fig. 2 to a damped sine wave. For a specific direction,

1 $$r={r}_{0}+A{{{{\rm{e}}}}}^{-{{\Gamma }}t}\sin (2\pi {\nu }_{{{{\rm{R}}}}}t+\phi )$$

where *r* is the sample’s radius, *r*_0_ is the sample’s average radius, *A* is the oscillation’s maximum amplitude, *t* is the oscillation time, Γ is the decay constant, *ϕ* is the phase shift, and *ν*_R_ is the resonant frequency.

**Table 1:** Parameters used in front tracking analysis.

Parameters	Air	Droplet
Density (kg m^−3^)	1.2	2900
Viscosity (Pa s)	1.9 × 10^−5^	0.045
Surface tension (N m^−1^)	-	0.65
Impact velocity (m s^−1^)	-	0.4

For the two simulated oscillation scenarios, there are no levitation forces interacting with the droplets and, therefore, the l = 2 mode’s resonant frequency will not split. This means the Rayleigh equation^21^ can be applied directly. For the l = 2 oscillation mode, surface tension *γ* is expressed as follows^21^:

2 $$\gamma =\frac{3}{8}\pi {\nu }_{{{{\rm{R}}}}}^{2}M$$

Here, *ν*_R_ is the resonant frequency and *M* is the sample’s mass. The calculated resonant frequency and surface tension based on the simulated droplet’s oscillations are listed in Table 2. Due to the numerical uncertainties in the simulation, it is normal for slight differences to be observed in the input and output surface tension. In addition, the front tracking method is based on the linear theory and is most suitable for simulating oscillations with very small amplitudes. Previous reports using FT simulation to study droplet oscillations^28–30^ commonly reported slight discrepancies of a few percent between the theoretical and output frequencies.

**Table 2:** Simulated Al_2_O_3_ droplet’s resonant frequency *ν*_R_ (Hz) and surface tension *σ* (N m^−1^) obtained with the front tracking simulation method.

	x-direction		y-direction		z-direction	
	*ν*_R_	*σ*	*ν*_R_	*σ*	*ν*_R_	*σ*
Freely oscillating	182	0.629	182	0.629	183	0.632
Drop-bounce	183	0.633	183	0.634	183	0.633

### Surface tension of molten Al_2_O_3_ measured with the drop-bounce method

The oscillation behavior of a bouncing molten Al_2_O_3_ sample, obtained using the drop-bounce method, is shown in Fig. 1. We can see that the oscillation of a bouncing droplet obtained via simulation and that obtained via experiment closely resemble each other. However, during the experiment, in cases where the sample moves closer to or away from the camera during its bouncing period, the following equation is used when fitting the damped oscillation curve:

3 $$r=({r}_{0}-\alpha (t))+\left(\frac{{r}_{0}-\alpha (t)}{{r}_{0}}\right)A{{{{\rm{e}}}}}^{-{{\Gamma }}t}\sin (2\pi {\nu }_{{{{\rm{R}}}}}t+\phi )$$

**Fig. 3:**
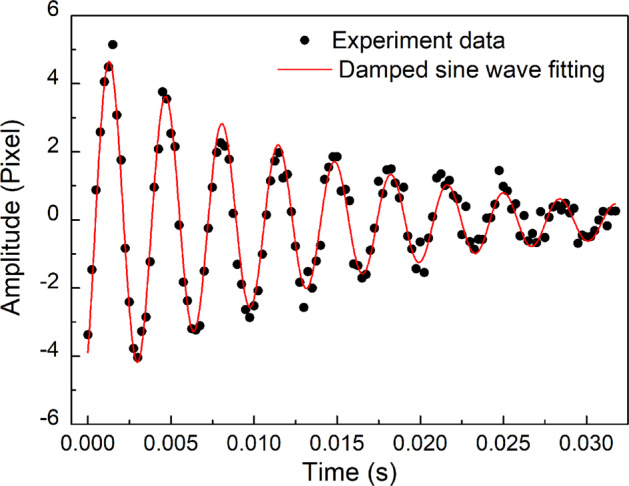
Damped oscillation along the horizontal axis of a bouncing molten Al_2_O_3_ droplet. The sample’s oscillation amplitudes while bouncing upward during the drop-bounce experiment are shown as black circles. Since the sample's surface can be considered as undergoing damped simple harmonic oscillation motion, least squares fitting using a damped sine wave gives the sample's resonant frequency required for surface tension calculations. The damped sine wave of best fit is shown in red.

**Fig. 4:**
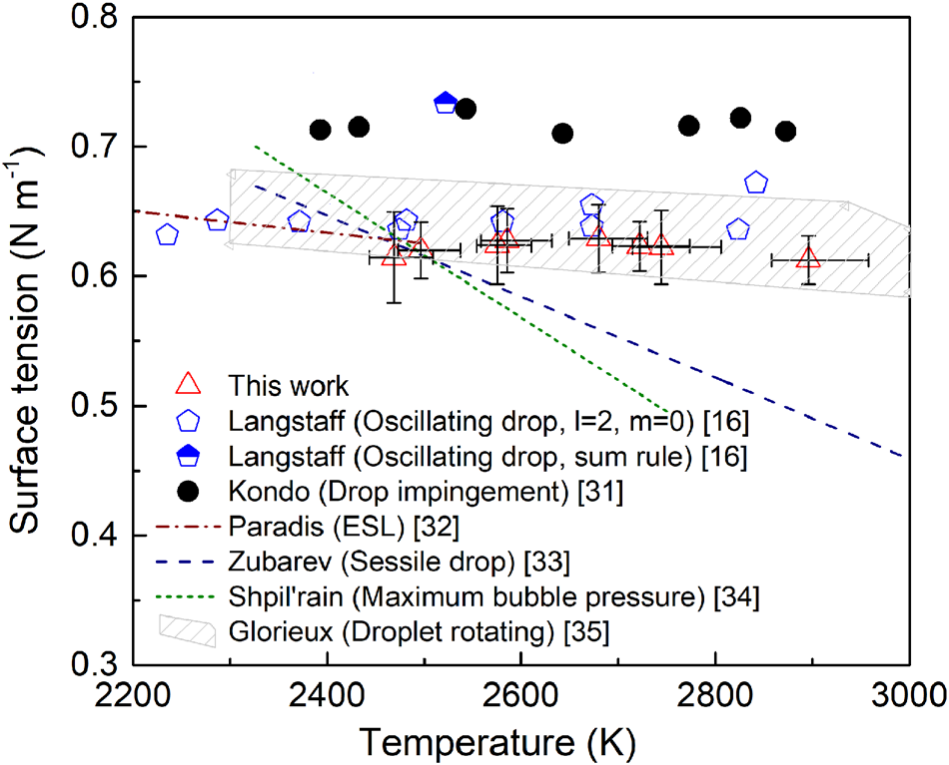
Surface tension of molten Al_2_O_3_. The surface tension of molten Al_2_O_3_ reported in this study with the drop-bounce method is shown in red triangles. The error bars for surface tension are standard uncertainties calculated based on the standard uncertainties of the sample's resonant frequency and mass. The error bars for temperature are its maximum and minimum values, calculated considering the effects of radiative and convective heat loss. Our data agree well with those from previous studies^16,32,35^ using contactless methods, which all suggest that the surface tension of molten Al_2_O_3_ is weakly temperature-dependent.

Here, *α*(*t*) is a time-dependent fitting parameter that describes the change in the observed sample’s radius caused by the sample moving towards or away from the camera. In the ideal case where the sample bounces upwards perfectly vertically, the *α*(*t*) term is zero and Eq. (3) simplifies to Eq. (1). The oscillation amplitude of the sample’s radius along the horizontal direction (*x*-axis) is shown in Fig. 3 along with the fitted damped sine wave. In general, data gathered from the sample’s oscillation along the *x*-axis is used because they are least affected by sample rotation. For an ellipsoid-shaped droplet, rotation along the horizontal *x*-axis and the vertical *z*-axis does not affect the observed sample’s *x*-axis oscillation amplitude when viewed in 2D. On the other hand, sample rotation along the horizontal *y*-axis (along the camera’s point of view) is easily noticed and discarded because it complicates the oscillation analysis. The combined standard uncertainty of the calculated surface tension, *u*_*γ*_, is evaluated using the following equation:

4 $${u}_{\gamma }^{2}={\left(\frac{\partial \gamma }{\partial {\nu }_{{{{\rm{R}}}}}}\right)}^{2}{u}_{{\nu }_{{{{\rm{R}}}}}}^{2}+{\left(\frac{\partial \gamma }{\partial M}\right)}^{2}{u}_{M}^{2}$$

and the combined standard uncertainty of the sample’s mass term, *u*_*M*_, is expressed as:

5 $${u}_{M}^{2}={u}_{{M}_{{{{\rm{evap}}}}}}^{2}+{u}_{{M}_{{{{\rm{scale}}}}}}^{2}$$

where ${u}_{{M}_{{{{\rm{evap}}}}}}$ is the standard uncertainty of the sample’s mass change due to evaporation, and ${u}_{{M}_{{{{\rm{scale}}}}}}$ is the standard uncertainty of the measured sample mass after the ADL experiments. When least squares fitting using Eq. (3) was applied to the experiment data, the standard uncertainty of the resonant frequency, ${u}_{{\nu }_{{{{\rm{R}}}}}}$, can be obtained. The standard uncertainty of each surface tension data point was calculated and represented as error bar in Fig. 4. A detailed example on how the combined standard uncertainty of surface tension is evaluated is provided in Table 3.

**Table 3:** Combined standard uncertainty evaluation for the surface tension of a random sample.

Source	Standard uncertainty	Sensitivity coefficient	Contribution
Uncertainty in the sample’s resonant frequency, ${u}_{{\nu }_{{{{\rm{R}}}}}}$	5.08 s^−1^	5.85 × 10^−3^ kg s^−1^	2.97 × 10^−2^ N m^−1^
Uncertainty in the sample’s mass, *u*_*M*_	6.69 × 10^−8^ kg	5.38 × 10^4^ s^−2^	3.57 × 10^−3^ N m^−1^
Evaporation of the sample, ${u}_{{M}_{{{{\rm{evap}}}}}}$	1.74 × 10^−8^ kg	5.38 × 10^4^ s^−2^	9.38 × 10^−4^ N m^−1^
Accuracy of the scale, ${u}_{{M}_{{{{\rm{scale}}}}}}$	6.41 × 10^−8^ kg	5.38 × 10^4^ s^−2^	3.45 × 10^−3^ N m^−1^
Combined standard uncertainty, *u*_*γ*_			3.00 × 10^−2^ N m^−1^

During the bouncing period, the sample is oscillating while experiencing free-fall and, therefore, Eq. (2) can be applied without modification. The surface tension of molten Al_2_O_3_ obtained in this study with the drop-bounce method are shown in Fig. 4 along with values obtained from the literature^16,31–35^. Data reported by Kondo et al.^31^ with the drop impingement method is slightly higher than the rest of the reference data. We believe this deviation could result from an overestimation in the sample’s kinetic energy that was used to generate additional surface areas and uncertainties in the calculated sample’s surface area at maximum deformation.

## Discussion

Based on the simulated results shown in Figs. 1 and 2 and Table 2, we can conclude that the oscillation of a bouncing sample is the same as an induced l = 2 mode oscillation onto a freely levitated sample. The agreement in the resonant frequencies between the two scenarios, as shown in Table 2, suggests that resonant frequency splitting does not occur for the bouncing droplets in drop-bounce experiments, which is expected considering the sample is oscillating during free-fall and experiencing microgravity.

As shown in Fig. 4, the surface tension of molten Al_2_O_3_ obtained with the drop-bounce method accords with that reported by Langstaff et al.^16^, Glorieux et al.^35^, and Paradis and Ishikawa^32^. Compared to these surface tension values of Al_2_O_3_ obtained with contactless methods, those by contact methods^33,34^ show a sharper decreasing trend with increasing temperature. Because surface tension is highly sensitive to any surface contamination, we believe contactless methods can produce more reliable data. As mentioned in the introduction, Langstaff et al. conducted oscillating drop experiments using a ground-based ADL setup and calculated the sample’s surface tension using only the primary resonant frequency of the l = 2 m = 0 mode with Eq. (2). Although it is considered as inaccurate to calculate surface tension without incorporating the spilt resonant frequencies, no correction terms are available for ADL. By linearly fitting and comparing the data from this work and by Langstaff et al., a maximum deviation of 6% was observed. This good agreement in the calculated surface tension of molten Al_2_O_3_ between the drop-bounce method used in this study and that reported by Langstaff et al. suggests that corrections to the resonant frequency, such as the sum-rule for EML, is not strictly necessary for ADL.

We consider this a key finding in the field of aerodynamic levitation experiments because it validates the surface tension data obtained using the oscillating drop method with ground-based ADL, something not possible prior to this study because of the lack of a “sum-rule.” As a well-established measurement technique, the oscillating drop method has several advantages over the newly developed drop-bounce method. For example, the oscillating drop method provides more accurate temperature measurements as the sample is monitored with a pyrometer throughout the experiment. Further, the sample is never in contact with any surface during the measurement, which means the surface tension of undercooled materials can be obtained with the oscillating drop method. However, regardless of the method used, noticeable scattering in the obtained surface tension data can be seen in Fig. 4 due to the difficulty of conducting experiments at extremely high temperatures (3000 K). Therefore, the availability of two measurement methods that can be used with ADL—the oscillating drop and drop-bounce methods—for surface tension analysis allows for better discussion on the accuracy of the reported data for molten oxides.

In summary, in this study, we showed that the l = 2 mode oscillation of a freely levitated droplet and the oscillation of a bouncing droplet in drop-bounce experiments are identical. In addition, the surface tension of molten Al_2_O_3_ obtained with the drop-bounce method matches the reference data obtained using the oscillating drop method with only the l = 2 m = 0 mode’s resonance frequency. Therefore, we can conclude that if a primary oscillation of the l = 2 m = 0 mode is induced with ADL, the droplet’s surface tension can be reasonably accurately calculated while ignoring the effect of the l = 2 m = ±1, ±2 oscillation modes. In other words, unlike the case for ground-based EML, ground-based ADL does not strictly require a “sum-rule” for surface tension calculations.

## Methods

### Front tracking method

The FT method is a well-established method for the direct numerical simulation of droplet or bubble deformation. The flow is accurately solved by the Eulerian mesh method, and the interface is precisely tracked by a set of movable surface nodes^36^. In particular, the FT method is suitable for simulating droplet collision or bouncing movements^37^. The governing equations are the mass conservation equation and the Navier–Stokes equation for fluids. Both the gas and liquid phases were considered in the simulations. The finite volume method is used to discretize the governing equations. Different fluids are treated as one material with variable density and viscosity. Specifically, the density and viscosity vary sharply across the interface. The interface separating different fluids is tracked by the front tracking method, where the interface is represented by a surface mesh (namely, a set of connected marker points). The curvature is precisely computed from the surface mesh, and the surface tension is considered as volumetric forces using the continuum surface force model^38^. The PArallel, Robust, Interface Simulator (PARIS) software application was used for the simulation. The implementation details of the method are clearly presented by Aniszewski et al.^39^.

The initial setup in our simulations was as follows. Because the l = 2 mode oscillation takes on an ellipsoid shape, in the free oscillation case, an initial ellipsoid was configured at the domain center to trigger the oscillation. The ellipsoid’s major and minor axis lengths were 2.6 mm and 2.0 mm, respectively. After deformation, the droplet diameter was 2.2 mm in the steady state. In the drop-bounce case, a spherical droplet with an initial vertical velocity of 0.4 m s^−1^ was configured just above the bottom wall boundary. To keep the droplet volume the same in both cases, the droplet diameter was set to 2.2 mm. Then, the droplet impacted the wall, bounced from the bottom wall, and oscillated in the air. Because the air effect was considered, the calculation domain had to be selected sufficiently large that the droplet behaviors were not significantly influenced by the domain sizes. Different calculation domain sizes were tested, and a domain of 10 mm × 10 mm × 10 mm was found to be sufficiently large to neglect the influence of the domain boundary in both cases. Meanwhile, the mesh size must be selected sufficiently fine to obtain the convergent behaviors of droplet oscillation. Different mesh sizes were therefore tested, and we found that a mesh size of 0.083 mm could provide reliable results in both cases. Specifically, the adopted mesh number corresponding to a mesh size of 0.083 mm was 120 × 120 × 120 in both simulations. After the simulation, the droplet oscillation behaviors in both cases were analyzed using Python.

### Drop-bounce method with aerodynamic levitation

**Fig. 5:**
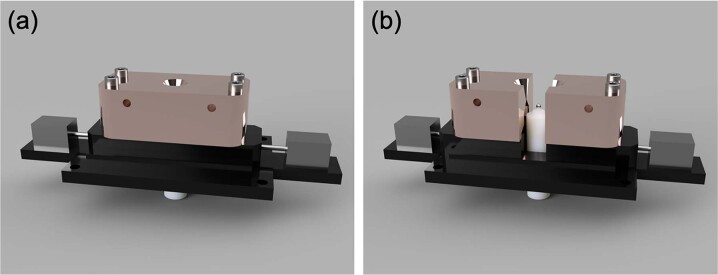
Simplified schematic of the splitable nozzle when closed and opened. **a** shows the closed splitable nozzle that is used for sample heating and levitation. **b** shows the sample free-falling after the nozzle splits open during the drop-bounce experiment. The sample eventually bounces off the boron nitride platform placed a few millimeters underneath the nozzle's orifice. The 3D models were constructed by Y. Sun.

A detailed description of the setup used in the drop-bounce experiments can be found in Sun et al.^26^. Some adjustments were made to the design owing to the use of different lasers for heating oxides. To drop the sample after it is levitated, a split-able nozzle was used; a schematic of the nozzle is shown in Fig. 5. The nozzle remains closed while levitating the sample, and opens the moment the laser is turned off to drop the sample. In this study, a 10.6 μm CO_2_ laser placed directly on top of the sample was used for heating. A 0.9 μm pyrometer (IR-CAS8CNL, CHINO) was used for monitoring the sample’s temperature. The pyrometer’s signal was collected by a data logger (midi LOGGER GL240, Graphtec) every 10 ms. To obtain a better contrast between the sample and the background, the shadowgraph imaging technique^40^ was adopted. A UV background was generated with a background light and a 370 nm bandpass filter. During the experiment, images were taken by a high-speed camera (MEMRECAM HX-7s, nac Image Technology) equipped with a telecentric lens (1.0X, 2/3”, Gold TL, Edmund) at a resolution of 1024 × 928 and 4000 fps. A camera shutter speed of 1/50000 s was used to ensure the recorded images were not too dark. In this study, the Al_2_O_3_ (4N powder, Kojundo Chemicals) samples were prepared using spark plasma sintering (SPS) at a temperature of 1623 K and a pressure of 100 MPa under argon flow (6N, Air Liquide). The prepared Al_2_O_3_ pellets were cut into smaller 5–10 mg pieces and melted into 1.5–2 mm diameter spheres. To prevent sample reduction, a mixture of Ar (6N, Air Liquide) and O_2_ (3N up, Taiyo Nippon Sanso) (7:3 flow ratio) was used to levitate the oxides. Negligible mass change (less than 0.1%) was observed in each of the Al_2_O_3_ samples before and after the drop-bounce measurements.

**Fig. 6:**
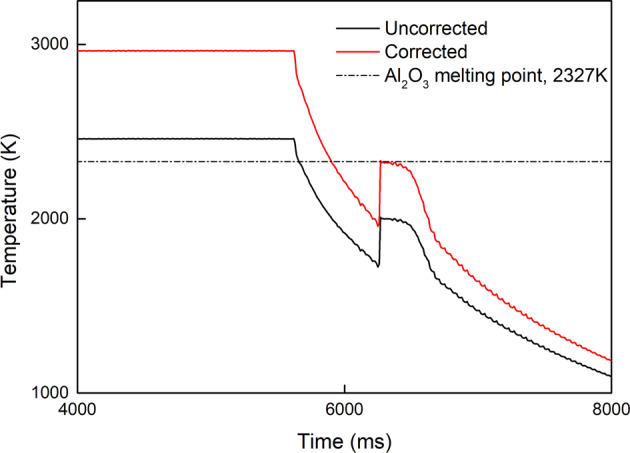
Uncorrected and corrected temperature curves of molten Al_2_O_3_. The uncorrected temperature curve, shown in black, is calculated under the assumption that the sample is a blackbody with an emissivity equal to one. Since the sample is not a blackbody, its emissivity must be corrected using its melting point and Wien's law to accurately reflect its temperature. The red curve is the corrected temperature curve where the temperature at the end of recalescence matches the known melting point of Al_2_O_3_.

The untreated temperature data collected from the pyrometer treat the sample as a blackbody with an emissivity equal to one. This must be corrected based on the sample’s melting point and a derivation of Wien’s law:

6 $$\frac{1}{T}-\frac{1}{{T}_{{{{\rm{pyro}}}}}}=\frac{1}{{T}_{{{{\rm{m}}}}}}-\frac{1}{{T}_{{{{\rm{m,pyro}}}}}}$$

where *T* is the corrected temperature; *T*_pyro_ is the temperature recorded by the pyrometer; *T*_m_ is the melting point of the sample; and *T*_m,pryo_ is the temperature recorded by the pyrometer at the sample’s melting point. *T*_m,pyro_ can be identified by the increase in the sample’s temperature due to recalescence after undercooling. A comparison between the uncorrected and corrected cooling curves for molten Al_2_O_3_ is shown in Fig. 6.

When using the drop-bounce method for surface tension measurements, the sample is no longer visible for the pyrometer after it is dropped. To evaluate the sample’s temperature during the drop-bounce period, we considered the following two scenarios. We first assumed that the sample’s heat loss behavior during levitation and during the drop-bounce period were the same. In this study, the time-dependent temperature curve of the levitated sample during free-cooling before solidification—like the free-cooling section while the sample is still in its liquid phase, shown in Fig. 6—was fitted with a polynomial equation. This polynomial equation was used to calculate the sample’s temperature during the drop-bounce period. This approach gives us the lower limit of the estimated temperature (overestimated heat loss) as the sample should experience more convective heat loss while levitated because of the levitation gas flow^26^. The upper limit of the estimated temperature is calculated by assuming the sample only experienced radiative heat loss without convection. The sample’s emissivity at 0.9 μm was used in the radiative heat loss calculation. In addition, the sample’s surface tension was determined based on its oscillation behavior as it cooled, approximately over a period of 50 ms (128 frames). Therefore, the upper temperature limit should be calculated using the time elapsed until frame no.1, and frame no.128 for the lower temperature limit. The upper and lower temperature boundaries are shown as error bars in Fig. 4.

**Fig. 7:**
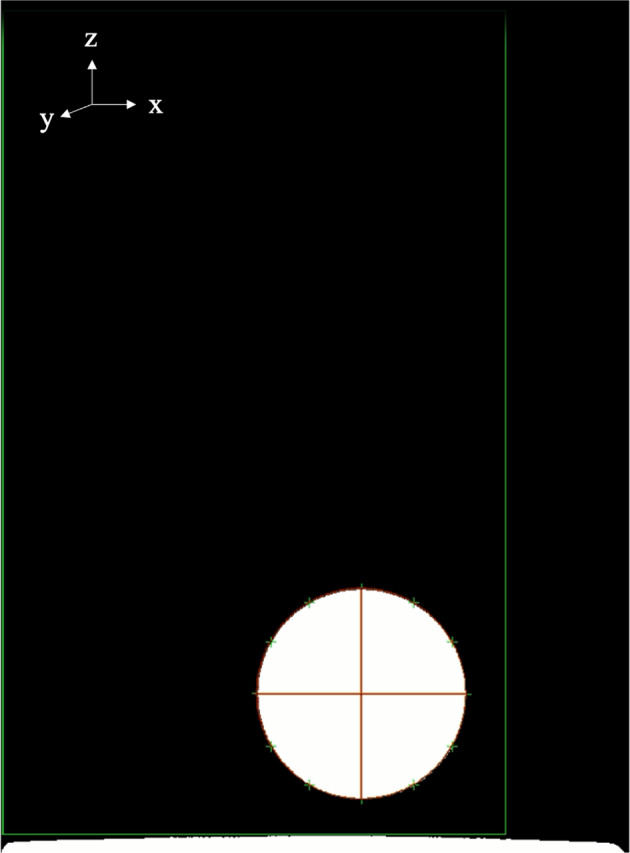
Elliptical fitting on the captured shadowgraph of the molten Al_2_O_3_ sample. The cross-section of a spherical sample undergoing a small-amplitude l = 2 m = 0 mode oscillation closely resembles that of an ellipse. Therefore, least squares fitting of the sample's contour with an equation for an ellipse is done using the software DIPP-Macro II. The red lines represent the sample's major and minor axis after fitting. The photograph was taken by T. Takatani.

The shadowgraph images recorded by the high-speed camera were processed using the image analysis software DIPP-Macro II. An image of a molten Al_2_O_3_ sample analyzed with DIPP-Macro II is shown in Fig. 7. The software performs edge detection, and 12 equally-spaced points (every 30°) along the detected edge were used for elliptical fitting. The sample oscillating during the bouncing period takes on an elliptical shape when viewed in 2D and the length of the horizontal axis can be extracted in pixels. Detailed information on the image analysis method can be found in a previous publication^26^.

### Reporting summary

Further information on experimental design is available in the Nature Research Reporting Summary linked to this paper.

## Supplementary information

Reporting Summary Checklist

## Data Availability

The data that support the findings of this study are available from the corresponding author, Y.S., upon reasonable request.

## Code availability

The code related to the front tracking simulation that supports the findings of this study is available from the corresponding author, Y.S., upon reasonable request.
